# Discordance between peritumoral and subareolar injections for mapping sentinel lymph nodes in the breast

**DOI:** 10.1007/s10549-024-07491-8

**Published:** 2024-09-15

**Authors:** Josephine Situ, Cameron Walker, Tharanga D. Jayathungage Don, Hiroo Suami, David K. V. Chung, Hayley M. Reynolds

**Affiliations:** 1 Department of Engineering Science, The University of Auckland, Auckland, New Zealand; 2 Auckland Bioengineering Institute, The University of Auckland, Auckland, New Zealand; 3 Australian Lymphoedema Education Research and Treatment Program (ALERT), Department of Health Sciences, Faculty of Medicine, Health and Human Sciences, Macquarie University, Sydney, NSW Australia; 4 Alfred Nuclear Medicine and Ultrasound, Newtown, NSW Australia; 5 Discipline of Child and Adolescent Health, Sydney Medical School, University of Sydney, Sydney, NSW Australia

**Keywords:** Breast cancer, Peritumoral, Sentinel lymph node, Subareolar

## Abstract

**Purpose:**

Sentinel node biopsy (SNB) is a common staging tool for breast cancer. Initially, peritumoral (PT) injections were used, however subareolar (SA) injections were later introduced to simplify the technique. Controversy remains regarding whether PT and SA injections map the same sentinel lymph nodes (SLNs). This study aimed to determine whether the regional location of breast SLNs differs when using PT versus SA injections using a large dataset from a single institution.

**Methods:**

A total of 1035 patients who underwent breast SNB (PT injections: *n* = 858 and SA injections: *n* = 177) with lymphoscintigraphy and SPECT/CT were included. The identified SLN locations using SA injections were compared with those using PT injections. Differences in drainage proportions and odds ratios (ORs) for each clockface breast region and the whole breast were calculated using a two-proportion z-test and Fisher’s Exact Test.

**Results:**

A higher proportion of internal mammary SLNs were identified using PT injections for the whole breast (0.30 versus 0.09) and for all breast regions, with all regions showing statistical significance except the upper outer quadrant. Similarly, ORs showed identification of internal mammary SLNs was significantly higher when using PT injections (4.35, 95% CI 2.53 to 7.95). There were no significant differences in identifying axillary SLNs between injection sites.

**Conclusion:**

This is the largest cohort study to compare the regional location of breast SLNs identified using PT injections versus SA injections. Discordance was shown in the SLNs identified between injection techniques, with PT injections more frequently identifying internal mammary SLNs.

**Supplementary Information:**

The online version contains supplementary material available at 10.1007/s10549-024-07491-8.

## Introduction

Lymphoscintigraphy (LS) and SPECT/CT is a standard imaging technique for mapping sentinel lymph nodes (SLNs) in breast cancer patients [1]. However, there have been ongoing controversies regarding which radioactive tracer injection site accurately identifies the potential cancer-bearing lymph node [1, 2]. Some support the use of superficial injections, which can involve subareolar (SA), periareolar, intradermal, and subdermal administration, while others support the use of deep injections, which include peritumoral (PT) or intratumoral delivery. Superficial SA injections have recently become popular for multiple reasons, including their fast SLN visualisation, lower use of resources as they do not require ultrasound guidance, and the fact that tracer at the injection site does not obscure axillary SLNs which can occur when using deep injection techniques [1, 3]. Despite these benefits, SA injections are based on old anatomical studies which claim the SA plexus collects all lymph fluid from the breast [4], which may not be accurate [4]. Furthermore, clinical and anatomical studies have shown the breast is not a single ectodermal unit that always drains to the anteropectoral axillary lymph nodes as previously thought [4–7].

To investigate the differences between superficial and deep injection techniques for breast SLN mapping, in 2014 Ahmed et al. [2] and Sadeghi et al. [8] carried out systematic reviews and meta analyses. Sadeghi et al. summarised 30 studies that assessed SLN locations of deep versus superficial injections in the same patients simultaneously or serially, while Ahmed et al. only analysed randomised controlled trials and cohort controlled studies which compared deep versus superficial injections, resulting in 13 studies for review. Analysis by Sadeghi et al. concluded there is fairly high and clinically acceptable concordance between superficial and deep injection methods, however they are clinically more successful if used together and can decrease the overall false negative rate. Meanwhile, Ahmed et al. concluded that superficial and deep injections are both suitable for identifying axillary SLNs, but identification of extra-axillary SLNs is significantly different between the techniques with deep injections identifying extra-axillary SLNs more often than superficial injections, particularly internal mammary SLNs [9, 10].

Many studies in the review by Ahmed et al. used blue dye, and only two compared PT and SA injections using radioactive tracer alone and SPECT/CT to localise breast SLNs [5, 10]. First, a multicentre study by Noushi et al. [10] involving 39 patients who had sequential SA injections followed by PT injections, and second, a single-centre study by Uren et al. [5] who analysed proportion differences in SLN locations from 737 patients undergoing PT injections and 75 patients having SA injections. Noushi et al. showed a high level of discordance between SA and PT injections of 56%, and reported that no patient showed identical SLNs in internal mammary nodes from both injection types. Meanwhile, when analysing drainage for SA injections relative to PT injections, Uren et al. observed a higher proportion of patients had drainage to the axillary level I anterior nodes and a significantly lower proportion had drainage to internal mammary nodes. More recently, Freebody et al. [11] reported a study conducted in 2019 involving 123 patients who were given simultaneous PT, SA, and subcutaneous injections to identify breast SLNs. They were able to locate axillary SLNs 98% of the time and reported identifying internal mammary SLNs in 13% of their patients. Importantly, they found the different injection methods could identify different axillary SLNs. They concluded that multisite injections are an optimal way to identify axillary SLNs and that deep injections are required for internal mammary drainage to be reliably identified.

Despite multiple studies reporting high discordance between SA and PT injections for extra-axillary SLN identification, SA injections continue to be used in recent clinical studies [12–14]. To address the continued lack of consensus, we aimed to statistically analyse a large dataset from the same centre as Uren et al. [6] to determine differences in the location of breast SLNs when using SA versus PT injections. This analysis advances further than previous studies by incorporating more refined breast region information and SLN localisations, including classification of the axilla into several subregions, due to the enhanced anatomical information from SPECT/CT. By analysing this data, we also aimed to improve understanding of lymphatic drainage of the breast.

## Methods

### Patient data

All patients who underwent breast LS and SPECT/CT at Alfred Nuclear Medicine and Ultrasound (ANMU) in Sydney, Australia between April 2018 and December 2022 were retrospectively identified. Patients who had past surgery or neoadjuvant therapy were excluded as past treatment can influence lymphatic drainage patterns [1]. Patients with multifocal or large breast tumours (diameter > 3 cm) were also excluded because it was unclear which PT injection site was the source of the lymph flow. SA injections were administered when the patient was scheduled for a prophylactic mastectomy, had ductal carcinoma in situ (DCIS), or tumours not visualised on ultrasound. There were 23 patients who had their SLNs mapped in both breasts, so to preserve data independence the data from only one breast was included in the statistical analysis by ordering patients by study date and alternately removing data from the left or right breast.

A total of 1035 breast cancer patients were included in this study, with patient characteristics summarised in Table 1. There were 858 patients (mean age 60.0 years) who received PT injections and 177 patients (mean age 53.7 years) who received SA injections. All patients who received SA injections were female, and the majority of patients who received PT injections were female (853, 99.4%) with only 5 male patients who had received PT injections included in the entire study cohort. The PT injection type was performed in the left breast for 448 (52.2%) of patients, and the right breast for the remaining 410 (47.8%) of patients. The SA injection type was performed in the left breast for 86 (48.6%) of patients and the right breast for 91 (51.4%) of patients.

**Table 1:** Patient characteristics

Characteristic	Injection type	
	Peritumoral	Subareolar
Number of patients	858	177
Age, mean (range)	60.0 (25.5–92.5)	53.7 (27.8–86.1)
Sex		
Female	853	177
Male	5	0
Injection side		
Left	448	86
Right	410	91

### LS and SPECT/CT imaging

**Fig. 1:**
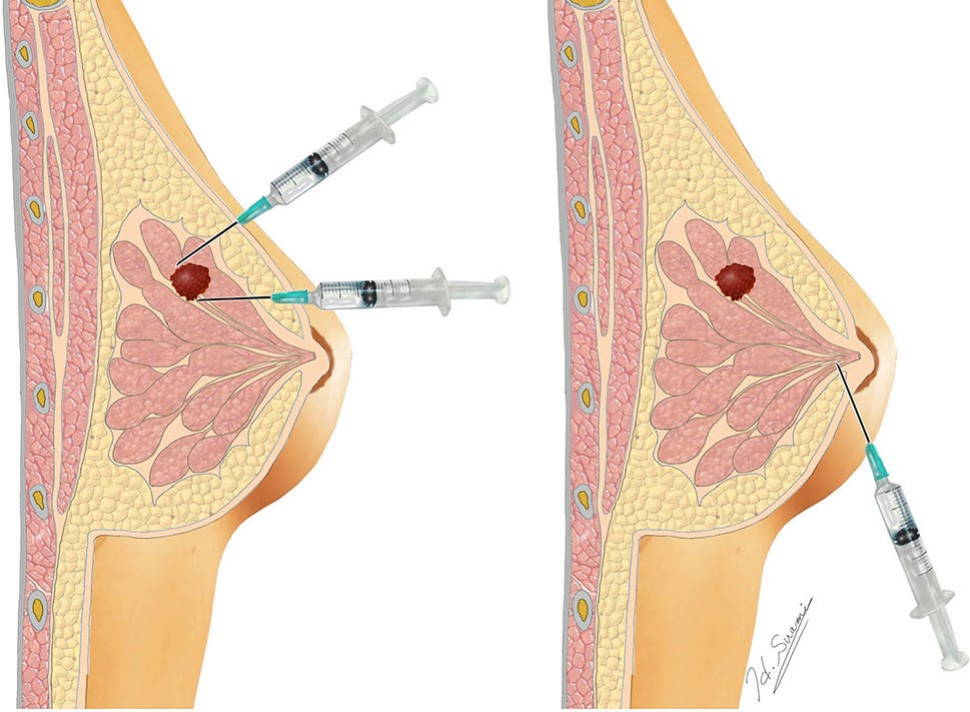
Peritumoral (PT) and subareolar (SA) injection types for administering the radiocolloid tracer during LS and SPECT/CT

The LS and SPECT/CT procedure at ANMU has been described in detail previously [6]. In brief, PT injections involved one to four injections (^99m^Tc-antimony sulphide colloid, each in 0.2 mL solution mixed with 0.2 mL 1% lignocaine) around the tumour under ultrasound guidance. For SA injections, two injections were delivered into the breast parenchyma under the areola from the upper outer breast quadrant. If drainage was poor, additional injections were made elsewhere in the breast parenchyma under the areola. The difference between PT and SA injection sites are illustrated in Fig. 1.

**Fig. 2:**
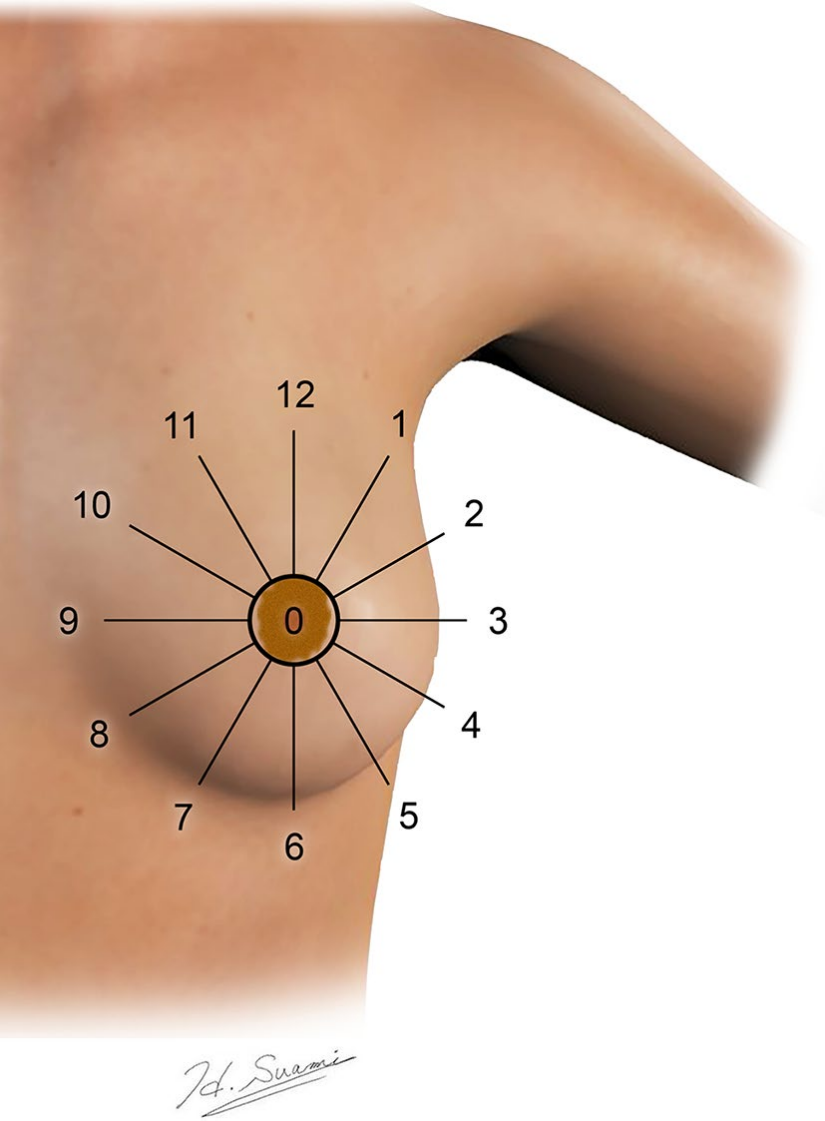
Schematic showing the standard 12 clockface regions in the left breast and the retroareolar 0 o’clock region

After the patient massaged the injection site for 5 min, LS was performed. If SLNs were identified on LS, SPECT/CT was acquired with the patient in the supine position. At the time of imaging, the nuclear medicine specialist manually annotated the position of SLNs on the SPECT images. The patient’s primary tumour was classified using the 12 standard regions according to the clockface system (Fig. 2). The distance of the tumour from the nipple was also recorded and if it was 1 cm or less, the tumour was reclassified as being within a new retroareolar 0 o’clock region. Patient SLNs were classified within one of 12 node fields, as described previously [6]: axilla level I (anterior), axilla level I (central), axilla level I (lateral), axilla level I (posterior), axilla level I (interpectoral), axilla level II, axilla level III, internal mammary, supraclavicular, mediastinal, interval, and contralateral. Interval nodes were defined as SLNs between the injection site and a standard draining node field and were most commonly intercostal or intramammary nodes. Further anatomical details about each node field location are given in Supplementary Table 1.

### Statistical analysis

The mapped SLN node field locations from each injection type were compared using a two-proportion z-test and Fisher’s Exact Test. The SLN locations when using PT injections were first compared with SA injections in each breast region, and then again by combining the SLN locations from PT injections across the entire breast. The two-proportion z-test was only performed if, for a particular node field (and for both injection types), there were at least five patients with and without drainage to that node field [15]. All p-values were Bonferroni-corrected [16] by multiplying by the number of comparisons. All statistical analyses were conducted using the R Statistical Package [17].

## Results

Table 2 shows results from the two-proportion z-test and Table 3 shows results from Fisher’s Exact Test. There were three node fields (mediastinal, contralateral, and supraclavicular) that were not tested as there was no drainage to these node fields with SA injections and rarely drainage with PT injections. An additional three node fields, axilla level I (lateral and interpectoral) and axilla level III could not be compared using the two-proportion z-test, as there were less than five SA patients with SLNs in these node fields. Combined axillary node fields had less than five SA patients without drainage, so these were not compared using the two-proportion z-test. Overall, almost all patients (99.1%) had an axillary sentinel node and 26.6% had an internal mammary sentinel node. Dual drainage to axillary and internal mammary nodes was common, involving 26.0% of patients. Patients with an internal mammary sentinel node but no axillary sentinel node was the smallest group, numbering 6 out of 1035 (0.6%) patients.

**Table 2:** Proportion of drainage to each node field, with PT injections separated into each clockface region including the retroareolar 0 o’clock region. Significantly different drainage between PT injections and SA injections are shown in bold font (Bonferroni-corrected *p*-value < 0.05)

		PT (breast region injection site, reflected to left side)													
Node field	SA	0	1	2	3	4	5	6	7	8	9	10	11	12	All regions
	*N* = 177	*N* = 108	*N* = 83	*N* = 198	*N* = 93	*N* = 56	*N* = 41	*N* = 31	*N* = 24	*N* = 23	*N* = 22	*N* = 56	*N* = 60	*N* = 63	*N* = 858
Axilla level I (anterior)	0.81	0.78	0.78	0.82	0.73	0.79	0.76	0.65	0.88	0.83	0.82	0.75	0.68	0.73	0.77
Axilla level I (central)	0.24	0.33	0.22	0.30	0.31	0.32	0.27	0.32	0.21	0.30	0.27	0.29	0.23	0.33	0.29
Axilla level I (posterior)	0.05	0.08	0.07	0.11	0.13	0.13	0.17	0.16	0.04	0.09	0.00	0.05	0.12	0.06	0.10
Axilla level II	0.06	0.13	0.11	0.07	0.08	0.05	0.07	0.00	0.08	0.13	0.05	0.09	0.07	0.13	0.08
Internal mammary	0.09	**0.33**	0.22	0.14	0.14	**0.29**	**0.39**	**0.52**	**0.71**	**0.65**	**0.45**	**0.50**	**0.48**	0.27	**0.30**
Interval	0.02	0.12	0.05	0.08	0.12	0.07	**0.24**	0.16	0.17	0.04	0.05	0.05	0.10	0.08	0.10

**Table 3:** PT odds (for a given breast region) divided by the SA odds, from Fisher’s exact test, with significant results given in bold font with associated 95% CIs (Bonferroni-corrected *p*-value < 0.05)

	Odds Ratio (breast region injection site, reflected to left side)													
Node Field	0	1	2	3	4	5	6	7	8	9	10	11	12	All regions
	*N* = 108	*N* = 83	*N* = 198	*N* = 93	*N* = 56	*N* = 41	*N* = 31	*N* = 24	*N* = 23	*N* = 22	*N* = 56	*N* = 60	*N* = 63	*N* = 858
Axilla level I (anterior)	0.83	0.86	1.11	0.65	0.87	0.74	0.43	1.66	1.13	1.07	0.71	0.51	0.64	0.80
Axilla level I (central)	1.56	0.86	1.32	1.41	1.48	1.14	1.48	0.82	1.36	1.17	1.25	0.95	1.56	1.28
Axilla level I (lateral)	0.54	1.43	0.59	0.63	0.00	0.00	1.93	0.00	0.00	0.00	1.05	0.98	0.94	0.68
Axilla level I (posterior)	1.92	1.65	2.64	3.13	3.02	4.35	4.06	0.92	2.01	0.00	1.20	2.79	1.43	2.32
Axilla level I (interpectoral)	2.10	1.62	1.12	0.95	0.79	2.22	0.00	0.00	0.00	0.00	0.00	0.73	1.42	1.09
*Axilla level I combined*	2.47	0.62	Inf	Inf	Inf	Inf	0.69	Inf	Inf	Inf	0.62	0.32	0.71	1.50
Axilla level II	2.49	2.03	1.17	1.36	0.95	1.32	0.00	1.52	2.50	0.80	1.64	1.19	2.43	1.53
Axilla level III	1.64	0.00	0.89	1.91	0.00	0.00	0.00	0.00	0.00	0.00	0.00	0.00	2.84	0.82
*Axilla levels I-III combined*	0.61	0.47	Inf	Inf	Inf	Inf	0.17	Inf	Inf	Inf	0.31	0.08	Inf	0.60
Internal mammary	**5.03 (2.52, 10.31)**	2.79	1.66	1.64	4.03	**6.44 (2.62, 15.60)**	**10.70 (4.08, 28.00)**	**24.40 (7.97, 78.80)**	**18.90 (6.23, 58.50)**	**8.39 (2.74, 24.80)**	**10.10 (4.54, 22.40)**	**9.41 (4.31, 20.70)**	3.72	**4.35 (2.53, 7.95)**
Interval	5.92	2.19	3.80	5.80	3.33	**14.00 (3.67, 63.60)**	8.32	8.65	1.97	2.06	2.45	4.81	3.73	4.63

The most common tumour site receiving PT injections was the 2 o’clock region (*n* = 198), closely followed by the retroareolar 0 o’clock region (*n *= 108). The least common tumour sites receiving PT injections were the 7, 8, and 9 o’clock regions (*n* = 24, 23, 22, respectively). Overall, Table 2 shows the proportion of SLNs identified in the internal mammary node field draining the entire breast was significantly higher when using PT injections compared to SA injections (0.30 versus 0.09). When analysing each breast region separately, there were nine regions with significantly higher proportions draining to the internal mammary node field when using PT injections compared to SA injections. This included the retroareolar 0 o’clock region, and regions from 4 o’clock through to 11 o’clock which together encompass the lower outer, lower inner and upper inner quadrants (Fig. 2). The upper outer quadrant, encompassing 1, 2, 3, and 12 o’clock regions, also had a lower drainage proportion to the internal mammary node field from SA injections, but none were statistically significant. The drainage proportion to interval nodes from PT injections was higher for all breast regions when compared with SA injections, but only significantly different for the 5 o’clock region (0.24 versus 0.02). No other drainage proportions had a statistically significant difference between injection types.

Odds ratios (PT odds divided by SA odds) in Table 3 show that for the entire breast the drainage to the internal mammary node field was significantly different between injection types, with an odds ratio of 4.35 (95% CI 2.53 to 7.95). When assessing odds ratios for each breast region, the internal mammary node field had eight breast regions with significantly higher odds ratios (ranging from 5.03 to 24.40). These included the retroareolar 0 o’clock region, and the 5 o’clock through to 11 o’clock regions, which also showed significant results in the proportions test (Table 2). The only difference between the two test results for the internal mammary node field was the 4 o’clock region did not have a significant odds ratio, but the 4 o’clock region proportion z-test results did show a significant difference between injection types. The interval node odds ratio was over 1.0 for all breast regions, but only significant for the 5 o’clock region (14.00, 95% CI 3.67 to 63.60). No other odds ratio value was statistically significant.

## Discussion

This study has statistically compared the regional location of breast SLNs identified by PT versus SA injections. The analysis has included the largest number of patients from a single-centre and was further strengthened by incorporating precise anatomical localisation of both breast tumours and SLNs from SPECT/CT. No other study has incorporated tumour locations discretised into clockface breast regions or had SLNs identified in as many axillary subregions as well as including interval nodes.

Findings showed there were no significant differences between the regional location of axillary SLNs identified by the injection types, even with multiple subregions including axillary level I (covering anterior, central, posterior, lateral, interpectoral groups), axillary level II, and axillary level III. This agrees with previous studies by Noushi et al. [10], although they grouped all axillary SLNs into one region, and Uren et al. [5], who did incorporate axillary subregions but did not discretise the breast into regions. Findings in this study also showed that identification of internal mammary SLNs was lower when using SA injections compared to PT injections, which was concordant with previous studies [2, 5, 10, 18], and was statistically significant in all clockface breast regions except those corresponding with the upper outer quadrant. The only other breast region with significant differences between injection sites was the 5 o’clock breast region, for identifying SLNs classified as interval nodes, which has not been reported previously. Interval nodes include intramammary and intercostal SLNs, which are often overlooked, even though they may have prognostic significance and show advanced pathological features when malignant [19].

Recent anatomical studies of the lymphatic system in the breast confirm these findings and support PT injections being more suitable than SA injections for accurately mapping SLNs [4]. Initially, SA injections were supported by historical anatomical studies that described lymphatics in the breast parenchyma converging to the subareolar plexus and then switching to the lymphatic vessels running to the axillary lymph nodes [20]. However, publications from the same group also depicted the lymphatic vessels in the upper torso connecting to the axillary lymph nodes but the relationship with these lymphatic vessels from two different origins was not clarified [4].

**Fig. 3:**
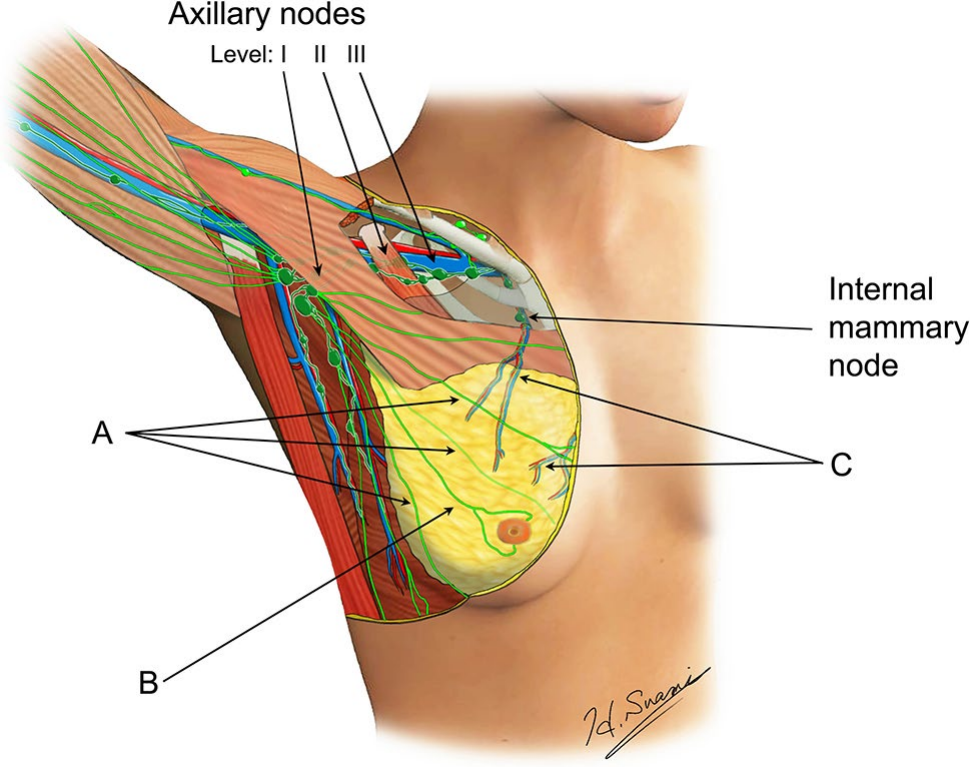
Lymphatic anatomy of the breast: **A** lymphatic vessels originating from the upper torso, **B** a lymphatic vessel originating from the areola and **C** perforating lymphatic vessels connecting to the internal mammary lymph nodes

Anatomical studies using a microinjection technique to visualise lymphatic vessels recently reported that lymphatics in the breast drain to the lymph nodes via three different lymphatic pathways [4]. This includes lymphatic vessels originating from the upper torso, lymphatic vessels originating from the areola, and perforating lymphatic vessels running with the perforating arteries to the internal mammary artery and connecting to the internal mammary lymph nodes (Fig. 3). This explains why PT injections are more likely to identify the internal mammary lymph nodes and may identify different SLNs from SA injections.

Metastatic involvement of internal mammary nodes is known to be higher in patients with medial tumours and positive axillary SLNs [21]. Furthermore, the metastatic status of internal mammary nodes is included in the American Joint Committee on Cancer staging criteria where it has prognostic importance similar to axillary node involvement [22] and undiagnosed internal mammary metastasis has been associated with significantly worse survival outcomes [23]. Therefore, ensuring accurate detection of SLNs in the internal mammary node field would logically be important for informing and optimising patient treatment, including surgery and radiation therapy options. If it is too risky to biopsy an internal mammary sentinel node, then the staging process will be incomplete and regional node irradiation to the internal mammary nodes should be considered. A recent review article by Lenihan et al. [24] reported four clinical trials which considered adjuvant internal mammary node irradiation when axillary nodes were positive. Overall, trial findings supported regional nodal irradiation including internal mammary nodes, with one Danish trial confirming it provided a survival advantage [25].

PT injections carried out in the retroareolar (0 o’clock) region and SA injection sites are both located behind the areola. However, PT injections are located at the depth of the tumour, which is deeper than SA injections located less than 0.5 cm from the skin surface. Hence, the statistical comparisons between retroareolar PT injections and SA injections suggest that deeper injections increase the drainage proportion to internal mammary node fields. These results contradict Noushi et al. [10], who found that the depth of PT injection did not have a significant effect on discordance, albeit with a smaller number of patients. The PT injection depth was not recorded in this study, and comparisons between patients may be difficult due to differences in breast shape and deformation. Further research to investigate the effect of injection depth on lymphatic drainage patterns is therefore warranted.

There are some limitations to this study, including the low number of patients with drainage in some breast regions, which meant the two-proportion z-test could not be carried out for all node fields. Additionally, SA injections were administered when patients were scheduled for a prophylactic mastectomy, had DCIS, or had tumours or DCIS not visualised on ultrasound. Consequently, SA patients were typically at a comparatively earlier cancer stage and were younger than PT patients (Table 1) which may impact lymphatic drainage, as studies by Kawase et al. [26], Kong et al. [27], and Lukesova et al. [28] have shown that younger patients have a higher likelihood of having internal mammary SLNs. Controlling for the effects of age may increase the difference between injection types for internal mammary drainage, but it was not carried out in this study. Furthermore, the number of injections and hence the volume of tracer varied between patients in both PT and SA groups. An increase in injection volume likely increases the drainage observed, so future analyses which take this into account would allow for greater confidence in the results.

## Conclusions

Lymphatic drainage from the breast to SLNs in the internal mammary node field is significantly more common when using PT injections than SA injections, in all regions of the breast excluding the upper outer quadrant. Significant differences persisted when restricting comparisons to retroareolar PT injections, suggesting injection depth is a factor impacting lymphatic drainage. There were no significant differences in lymphatic drainage to axillary subregions between the injection types. These results support the use of PT injections for accurate identification of breast SLNs, especially extra-axillary SLNs, and help improve understanding of breast lymphatic drainage patterns.

## Supplementary Information

Below is the link to the electronic supplementary material.

Supplementary file1 (DOCX 146 KB)

## Data Availability

The data analysed in this study are not publicly available due to ethical requirements but are available from the corresponding author on reasonable request.
