# Supplementary material for: Discordance between peritumoral and subareolar injections for mapping sentinel lymph nodes in the breast
Source: Breast Cancer Res Treat. 2024 Sep 15;209(2):283–90. doi: 10.1007/s10549-024-07491-8 (PMC11785644; doi:10.1007/s10549-024-07491-8)
Supplement: Supplementary file 1 — Supplementary file1 (DOCX 146 KB) [file 10549_2024_7491_MOESM1_ESM.docx]

**Supplementary material**

| **Node Field** | **Description** |
| --- | --- |
| Axilla Level I (anterior) | Below and lateral to the pectoralis minor muscle, along the lateral border of the pectoralis major muscle near the lateral thoracic artery |
| Axilla Level I (medial) | Below and lateral to the pectoralis minor muscle, lying at the mid base of the axilla in the axillary fat |
| Axilla Level I (lateral) | Below and lateral to the pectoralis minor muscle, lying on the lateral wall of the axilla, medial and posterior to the axillary vein |
| Axilla Level I (posterior) | Below and lateral to the pectoralis minor muscle, close to the posterior wall of the axilla near the subscapular artery |
| Axilla Level I (interpectoral) | Between the pectoralis major and pectoralis minor muscles |
| Axilla Level II | Behind the pectoralis minor muscle |
| Axilla Level III | Above the pectoralis minor muscle |
| Internal Mammary | In the internal mammary chain |
| Supraclavicular | Above the clavicles |
| Mediastinal | In the mediastinum |
| Interval | Between the tumour location and a standard draining node field, most commonly intercostal or intramammary nodes |
| Contralateral | SLNs on the opposite side of the body to the primary tumour location |

**Table S1:** SLN classifications and locations adapted from Uren et al. [6]
